# Recent Progress in Metal Nanowires for Flexible Energy Storage Devices

**DOI:** 10.3389/fchem.2022.920430

**Published:** 2022-05-24

**Authors:** Junxiang Wang, Wenxiang Piao, Xuanzhen Jin, Long Yi Jin, Zhenxing Yin

**Affiliations:** ^1^ Department of Chemistry, National Demonstration Centre for Experimental Chemistry Education, Yanbian University, Yanji, China; ^2^ Yanbian Zhenxing Electronic Technology Co., Ltd., Yanji, China

**Keywords:** flexible battery, flexible supercapacitor, copper nanowire, silver nanowire, gold nanowire

## Abstract

With the rapid evolution of wearable electronics, the demand for flexible energy storage devices is gradually increasing. At present, the commonly used energy storage devices in life are based on rigid frames, which may lead to failure or explosion when mechanical deformation occurs. The main reason for this phenomenon is the insufficient elastic limit of the metal foil current collector with a simple plane structure inside the electrodes. Obviously, the design and introduction of innovative structural materials in current collectors is the key point to solving this problem. Several recent studies have shown that metal nanowires can be used as novel current collector materials to fabricate flexible energy storage devices. Herein, we review the applications of metal nanowires in the field of flexible energy storage devices by selecting the three most representative metals (Au, Ag, and Cu). By the analysis of the various typical literature, the advantages and disadvantages of these three metal nanowires (Au, Ag, and Cu) are discussed respectively. Finally, we look forward to the development direction of one-dimensional (1D) metal nanowires in flexible energy storage devices and show the personal opinions with a reference value, hoping to provide the experience and ideas for related research in the future.

## Introduction

With the rapid expansion of smart electronics and communication technologies, the demand for wearable devices is gradually increasing, and the development of their flexibility, integration, and intelligence has become a new direction (Zhang X. et al., 2021). At the same time, the implantable medical devices have also attracted great attention as health awareness improves. Against this backdrop, there is an urgent need for electronic products that can be truly bent, folded, and stretched. To fully realize these flexible electronic products, the well-matched flexible energy storage devices are essential to be fabricated (Chen et al., 2017). However, the exploitation of flexible energy storage devices for wearable electronics has always been a tremendous obstacle to be overcome (Koo et al., 2012). As is well known, the typical electrochemical energy storage devices mainly include batteries (Dunn et al., 2011; Larcher and Tarascon, 2015) and supercapacitors (Simon and Gogotsi, 2008). As an important component of them, the electrode, which consists of the current collector and the active material, is a critical factor in determining the electrochemical performance and mechanical properties of energy storage devices.

Due to the nature of the material itself, unfortunately, most of the active materials for energy storage have a relatively low electrical conductivity and poor flexibility. When these active materials are coated on a planar current collector in a conventional manner, the large coating thickness and limited contact area make electrons react only with the surface part, which prevents the active material from fully displaying its capacity (Mai et al., 2014). More importantly, the difference in material and structure of the upper and lower layers caused by this coating method are extremely unfavorable to the mechanical deformation of the energy storage devices. When complicated deformation occurs, the active materials are easy to separate and fall off from the current collector, owing to the inability to maintain good contact between materials, resulting in a sudden drop of electrochemical performance or safety accidents caused by internal short circuit (Mo et al., 2017). Excitingly, the application of metal nanowires in electrodes can effectively help the active materials to exhibit their excellent electrochemical performance and remarkable flexibility (Chen et al., 2018; Kausar, 2019). The metal nanowires can be dispersed uniformly in the active material, like plant roots implanted in the soil, and are in complete contact with the active material. In theoretical, the “implanted” metal nanowires not only provide a pathway for the transfer of electrons so that the active material can be used to its full capacity, but also act the force-bearing framework, which can effectively relieve the stress generated during mechanical deformation.

**FIGURE 1:**
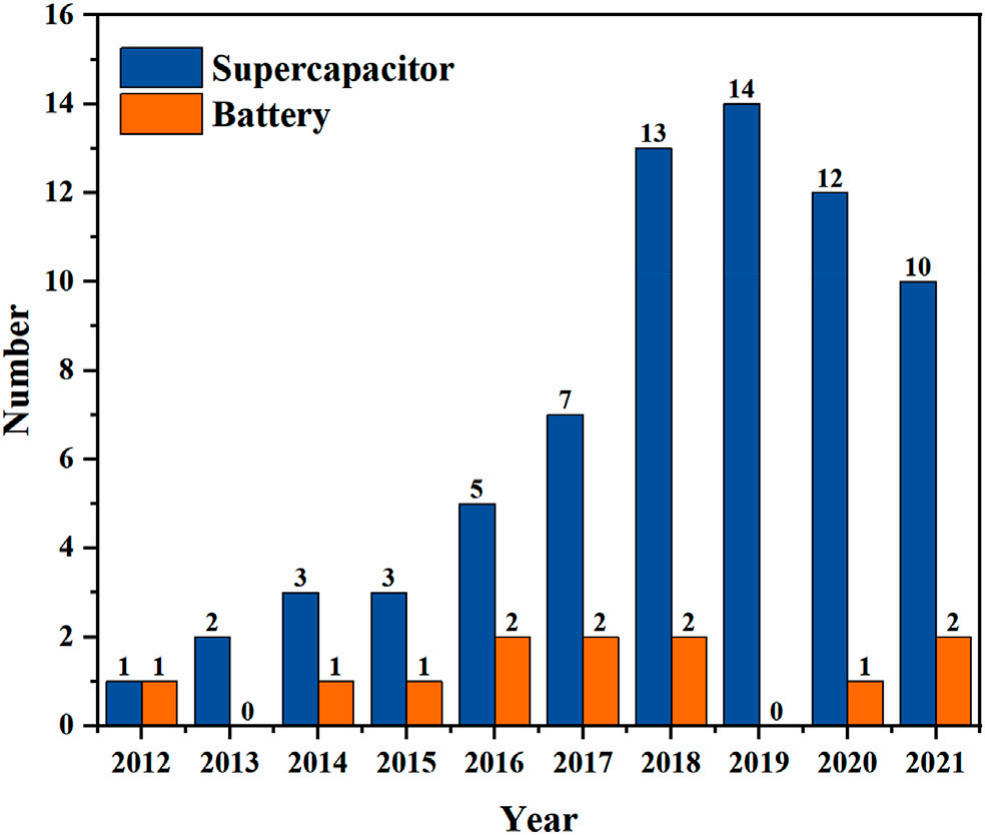
Trends in the number of publications on metal nanowire-based current collector for flexible energy storage devices over the past 10 years.

In the last decade, the metal nanowires have been widely used in the field of flexible electrodes and flexible electronic screens. With the continuous research, however, the application area of metal nanowires has been gradually broadened to the field of flexible energy storage devices. The research trend of metal nanowires for flexible energy storage in the last decade is shown in Figure 1. Therefore, for the future field of flexible electronics, the research of one-dimensional (1D) metal nanowires is undoubtedly very meaningful. In this paper, the applications of three representative 1D metal nanowires (Au, Ag, and Cu) in flexible energy storage devices (batteries and supercapacitors) are illustrated from the perspective of the material components, and their advantages and disadvantages are discussed. Furthermore, we look forward to the development of 1D metal nanowires, hoping to provide experience and ideas for the preparation of new electrochemical energy storage devices.

## Metal Nanowires

Metal nanowires have become a hotspot in many fields, due to their excellent electrical, thermal, optical, and mechanical properties. At present, metal nanowires are mainly used in conductive adhesives (Li et al., 2018), transparent conductive films (Zhou et al., 2020), sensors (Swetter et al., 2005), electrocatalysis (Ma et al., 2016), electrochemical energy storage devices (Mu et al., 2021), thermoelectric materials (Huang et al., 2014), etc. Most metal nanowires are prepared by solution methods, which are more economical and environmentally friendly than the preparation process (melting, forging, stamping, etc.) of bulk metals.

### Gold Nanowires

**FIGURE 2:**
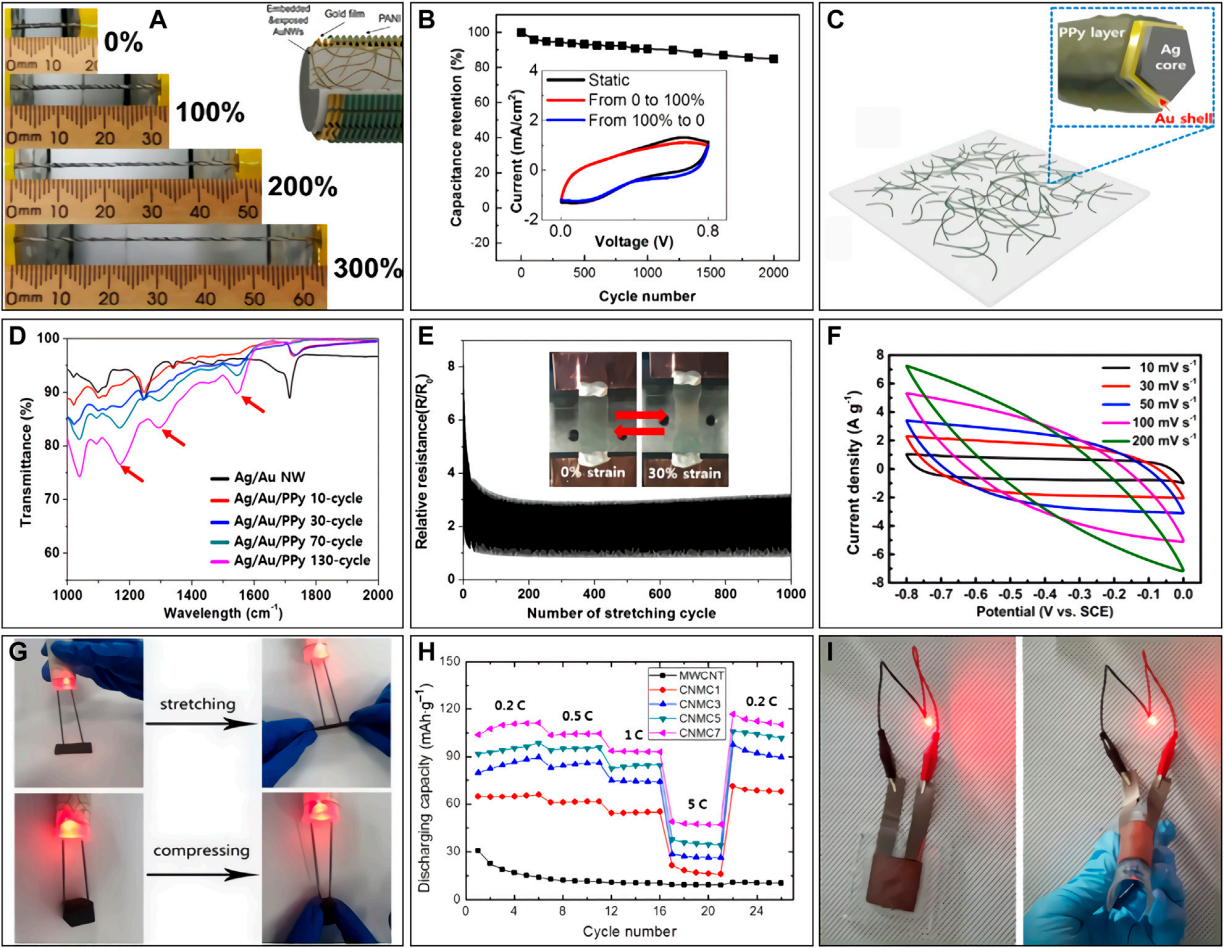
**(A)** Photographs of a stretchable fiber-based supercapacitor at strains of 0, 100, 200, and 300%, respectively. The inset images show the schematic illustration for the structure of the Au NWs/Au Film/PANI fiber electrode. **(B)** Capacitance retention of the stretchable fiber-based supercapacitors during 2000 cycles of 0%-200%-0% stretching/releasing. The inset shows the CV curves of the stretchable fiber-based supercapacitors in static status (black curves), stretching from 0% to 100% strain (red curve) and releasing from 100% to 0% (blue curve) at a fix scan rate of 100 mV s^−1^. Adapted from Zhao et al. (2018) with permission. Copyright 2018 American Chemical Society. **(C)** Ag/Au/PPy core-shell NW mesh film produced by electropolymerization of pyrrole. **(D)** FT-IR spectrum of Ag/Au and Ag/Au/PPy core-shell NW samples with different amounts of PPy. **(E)** The strain-dependent relative electrical resistance of Ag/Au/PPy core-shell NW mesh for 1,000 stretching cycles. Insets show the digital image of the electrical resistance measurement test of Ag/Au/PPy core-shell NW on a polydimethylsiloxane (PDMS) substrate. Adapted from Moon et al. (2017) with permission. Copyright 2017 Springer Nature. **(F)** CV curves of the as-fabricated supercapacitors were recorded at different scan rates ranging from 10 to 200 mV s^−1^. **(G)** Lighting photo of an LED with an external power supply connected by the conductive Cu NWs-MWCNT foam during stretching and compression. Adapted from Wang et al. (2021) with permission. Copyright 2021 Elsevier B.V. **(H)** The rate capability of Cu NWs-MWCNT and MWCNT anodes in the full cell with a polypropylene (PP) separator, LiPF_6_ electrolyte, and LiFePO_4_/MWCNT cathode. **(I)** Red LED powered by a flexible LIB. Adapted from Yin et al. (2018) with permission. Copyright 2018 Springer Nature.

Although gold nanowires (Au NWs) are not widely used in real life due to their high cost, it is essential to study the potential applications of Au NWs in energy storage from the perspective of scientific research. Recently, the application of Au NWs in the field of flexible supercapacitors is expanding. Importantly, the single Au NWs as supercapacitor electrodes cannot provide sufficient capacitance, so they need to be combined with high-capacitance materials (e.g., conductive polymers and metal oxides) to improve the capacitance of the prepared electrodes. In 2013, Chen et al. synthesized MnO_2_ spines grown on the Au NW stems (Chen et al., 2013). The high electrical conductivity of Au NW stems compensates for the low electrical conductivity of amorphous MnO_2_, thereby accelerating the redox reaction rate. When the Au NWs grew on the flexible polyethylene terephthalate (PET) substrate, the supercapacitors have good flexibility that can be freely bent in the range of 0°–180°. The test results show the composites have excellent electrochemical properties and good flexibility, which provide a research direction for the preparation of energy storage devices with better performance. In 2018, Zhao et al. reported a supercapacitor electrode based on Au NWs/Au Film/polyaniline (PANI) fiber with a double-helical winding design (Zhao et al., 2018). Although these electrodes exhibited a high stretchability (360%) and good capacitance retention (85% of capacitance after 2000 stretching cycles), as shown in Figures 2A,B, the low capacitance of supercapacitors (16.80 mF cm^−2^) still cannot meet our needs for widespread use in daily life.

In some applications, such as wearable devices and implantable devices, it requires electrode materials with high capacitance, flexibility, and transparency. Wang et al. synthesized Au NWs by solution method and prepared vertically aligned Au NWs by self-assembly bundling method (Wang et al., 2018). The prepared materials can achieve conductivity of 1.7 ± 0.8 Ω·sq^−1^ (low resistance) and high optical transparency of 90.3% at 550 nm. The symmetric supercapacitor (a thickness of ∼100 μm) can withstand up to 100% tensile strain with capacitance retention of 76%. Also, the maximum energy density of the supercapacitor is up to 3.8 mWh·cm^−2^ at a power density of 0.43 mW cm^−2^. Additionally, it exhibits excellent mechanical flexibility by repeatedly stretching (0–100% strain) and bending (0°–180°). Due to the excellent electrical conductivity and stability of Au NWs, the electrochemical performance of energy storage devices can be greatly improved. However, because of the high cost of raw materials for preparing Au NWs, Au NWs currently have practical applications only in high-tech fields such as biomaterials or aerospace.

### Silver Nanowires

Recent studies have found that silver nanowires (Ag NWs) have shown excellent utility in electronic device applications due to their excellent electrical conductivity. As we all know, the price of Ag is obviously lower than that of Au, so it is more widely used in daily life. Ag NWs are sought after in many fields because of their unique optical, electrical, mechanical, and biological properties (Guo et al., 2015; Jiang et al., 2019; Zhang et al., 2019; Zhang Y. et al., 2021). In the past decade, the application of Ag NWs in flexible transparent electrodes has been extensively studied (Han et al., 2021). Therefore, Ag NWs have gradually become a research hotspot of flexible conductive materials, which is considered one of the development directions of electronics in the future. Recently, the Ag NWs have shown great potential in flexible electrochemical energy storage devices (supercapacitors and batteries) has been demonstrated. Moon et al. prepared Ag-Au core-shell NWs and then coated the surface with polypyrrole (PPy) by electrochemical deposition (Moon et al., 2017). The Au shell not only protects the Ag core from oxidation but also changes the redox potential so that PPy is plated on the surface of the core-shell NWs. The Ag core NWs with optimal conductivity can increase the electron transfer rate, while PPy can provide high capacitance for the supercapacitor. Meanwhile, the prepared supercapacitor also has high transparency and flexibility (Figures 2C–E). However, the complicated fabrication process and high cost greatly limit its practical application. Li et al. prepared a pseudoplastic nanocomposite gel composed of Ti_3_C_2_T_x_-MXene nanosheets, manganese dioxide nanowires (MnO NWs), Ag NWs, and fullerenes, which fabricated the thick honeycomb porous forked-finger stretchable micro supercapacitor (MSC) by three-dimensional (3D) printing and unidirectional freezing methods (Li et al., 2020). The presence of a highly conductive 3D Ag NW network ensures fast and efficient charge transfer inside the electrode, further improving the electrochemical performance and efficiency. The internal honeycomb porous structure provides a degree of stability that allows the printed electrodes to withstand large deformations without fracture and significant performance degradation. When injected with a polymer gel electrolyte, the 3D-printed MSC exhibited extraordinary area capacitance (216.2 mF cm^−2^ at a scan rate of 10 mV s^−1^), energy density (19.2 µWh cm^−2^), and power density (58.3 mW cm^−2^), all of which are significantly higher than previously reported stretchable MSCs (Lee et al., 2019). Notably, at up to 50% stretching strain, the final MSC had an area capacitance reduction of less than 20% and maintained 75% of the initial capacitance after 1,000 stretch/release cycles. Finally, this stretchable MSC can be directly printed in 3D series and parallel modes, which has potential for application in practical wearable electronics.

With the increasing demand for multi-functional electronic devices, it is foreseeable that multi-functional flexible energy storage devices are one of the mainstream directions in the future. Wei et al. developed a novel electrochemically driven haptic sensor-based asymmetric supercapacitor (Wei et al., 2021). The cross-linked honeycomb CoV_2_O_6_/reduced graphene oxide (rGO) nanosheets prepared by electrodeposition and 1D Ag NWs integrated with elastic graphene aerogel framework (Ag NWs/GA) were used as positive and negative electrodes respectively. The Ag NWs/GA of the negative electrode can withstand different compressive strains and has good electrical conductivity due to its unique cross-linked structure and the presence of Ag NWs. Taking advantage of the excellent electrochemical properties of CoV_2_O_6_/rGO and the excellent elasticity of Ag NWs/GA, the authors integrated the sensor and supercapacitor to fabricate a novel stressed supercapacitor. The test results show that the multifunctional supercapacitor has a stable and fast current response to additional load strains from 10% to 90% and can be self-driven. It has broad application prospects in the fields of smart sensors, advanced bioelectronic devices, human-computer interaction, and electronics. At present, the combination of Ag NWs with other high-capacity materials is becoming very popular. Due to the unique properties of Ag NWs, it is expected to be used in future energy storage devices for wearable devices.

### Copper Nanowires

Copper nanowires (Cu NWs) are more widely used than other precious metals (Au and Ag) due to their abundant reserves and significantly lower prices. In addition, the excellent electrical conductivity (second only to Ag) makes the Cu NWs dominant in applying electrical devices. However, Cu is more reactive than Au and Ag, which makes it susceptible to oxidation under environmental conditions, resulting in a significant decrease in the electrical conductivity of Cu NWs. Therefore, improving the stability of Cu nanowires in applications is a hot research topic. Wang et al. synthesized Cu NWs by hydrothermal method and coated them on ethylene-vinyl acetate (EVA) foam by mixing Cu NWs and multi-walled carbon nanotubes (MWCNTs) in a specific ratio (Wang et al., 2021). The prepared stretchable/compressible conductive foam had high electrical conductivity (*R* = 2.40 Ω) and high mechanical stability (*R/R*_0_ < 1.6, after 100 cycles). Based on this, the commercial activated carbon was coated on the conductive foam to fabricate a flexible electrode. Figures 2F,G show the stretchable/compressible electrode with high capacity (124.3 F g^−1^) and high cycling stability (85.97% of capacitance retention after 500 cycles) was superior to commercial nickel foam. It is expected to be applied to flexible (stretching and compressing) supercapacitors and joint parts of bionic robots.

So far, commercialized wearable devices cannot achieve true flexibility, because the rigidity of traditional batteries is one of the biggest obstacles. How to achieve the flexibility of battery has become a hot topic of research. Huang et al. successfully fabricated a flexible high-performance potassium battery, which consisted of double-layered copper phosphide/Cu NWs (CuP_2_/Cu NWs) as the anode and perovinyl-3,4,9,10-tetracarboxylic dianhydride/carbon nanotubes (PTCDA@CNTs) as the cathode (Huang et al., 2021). The coin-type full cell exhibited a superior capacity of 117.3 mAh g^−1^ at 12,000 mA g^−1^ and good retention (reaching 80% at 400 mA g^−1^ after 842 cycles). In addition, the soft pack battery still maintained an ultra-stable open-circuit voltage after 5,000 cycles at a bending radius of 1.2 cm, and lit up LEDs at different folding angles, fully demonstrating its flexibility and stability. It provides directions for further improving the energy density and safety of wearable potassium ion batteries at ultra-high reaction rates. Chang et al. report a flexible, adhesive-free inorganic bilayer nanowire mesh electrode consisting of two nanowire fabrics of germanium nanowires (Ge NWs) and Cu NWs (Chang et al., 2017). When used as an anode in lithium-ion batteries, it provides high area capacity, mass capacity, and volume capacity. The combination of high theoretical capacity material (Ge NWs) and inactive material (Cu NWs) will deform spontaneously at the interface, creating good adhesion while maximizing the energy density of the electrode. The inactive Cu NWs network with low resistance can serve as a good substrate to support the broken Ge NWs and provide a fast electron transfer channel. Thus, the bilayer nanowire mesh electrode proposes another strategy for the electrode design of ultra-light and ultra-thin flexible Li-ion batteries with high capacity. Yin et al. prepared a novel lightweight 3D composite anode for fast charge/discharge lithium-ion batteries using the Cu NWs and MWCNTs as shown in Figures 2H,I (Yin et al., 2018). Through the welding process of the Cu NW network, the contact resistance of the electrode is effectively reduced without the usage of adhesives and conductive agents. In addition, the 3D Cu NW network structure in the electrode provides a rigid framework that not only prevents MWCNT from shrinking and swelling due to aggregation and expansion, but also minimizes the influence of volume changes during charging and discharging. The authors overcome the limitations of MWCNT as anode materials for fast charging/discharging lithium-ion batteries by using Cu NWs for the first time. The flexible lithium-ion batteries prepared by this composite anode maintained a capacity of >92.8% after 1,000 bends (radius 10 mm). This innovative anode structure could not only facilitate the manufacturing of ultra-fast charging electric vehicles, but also promote the development of flexible and wearable electronics.

Among the various metal nanowires, Cu NWs are considered to be the most promising metal nanowires for energy storage. Cu NWs can prepare composites with simultaneously high electrical conductivity, high capacity, and high cycling stability. At present, the most important problem limiting the development of Cu NWs is that Cu NWs are susceptible to oxidation, resulting in the attenuation of electrode capacity. Therefore, preventing the oxidation of Cu NWs and increasing the capacity of composites are the future research directions for Cu NWs-based energy storage devices.

## Perspective and Future

In terms of wearable and implantable electronics, the existence of flexible energy storage devices enables the electronics to fit well to human limbs and the internal structure of the body, which is a great alternative to traditional ones. Additionally, flexible energy storage devices can be combined with conventional fabrics to provide the energy for exoskeletons or other devices in the military field. It is expected that the metal nanowires are applied to produce a fully flexible battery with no fixed shape, which accommodates a higher degree of deformation and is promising to be used in electric vehicles to improve safety during a traffic collision. Also, these non-fixed shape power supplies can help wearable and implantable devices to be fully flexible, and improve medical diagnostics and treatment. Due to the nanoscale dimensions, the metal nanowires can be well dispersed in the solution that can be sprayed or spin-coated on surfaces with complex geometries to form thinner and purer films. This advantage has greatly facilitated their actual applications. From the viewpoint of the above-mentioned three metal nanowires, the Cu NWs have the most potential in flexible electrochemical energy storage, because of their high-cost effectiveness. Not only that, when applied in lithium batteries, the Cu NWs can form a “cage-like” metal network framework (anode) that suppresses volume changes and internal dendrite growth of Li during charging and discharging. However, the shortcoming is its poor chemical stability, so preventing the oxidation of Cu NWs is a current research hotspot.

As far as stability and cost-effectiveness are concerned, moreover, aluminum nanowires (Al NWs) with lightweight are certainly more promising than the Cu NWs owing to their dense oxide films and large crustal reserves. Most importantly, the Al NWs current collector (cathode) can be used in combination with the Cu NWs current collector (anode) to fabricate complete flexible lithium batteries, and the actual capacity of batteries can be increased by enhancing the contact area with active materials, which is attributed to providing more electron transport paths. For the development of new flexible energy storage devices, such as sodium batteries, the introduction of Al NWs holds promise for reducing the overall mass and thus boosting the energy density and power density. However, the safety requirements for the preparation process of Al NWs are difficult to meet due to the inherent high activity of Al. At present, the most effective strategy is to decrease the specific surface area of Al NWs, which effectively dropped the activity by adjusting the surface atomic ratio. Unfortunately, the excessive approach may lead to reducing the contact area between the Al NWs and the active materials, resulting in the low electrochemical performance of batteries. Therefore, we believe that the synthesis of Al NWs with the appropriate size is an important research direction in the future, which is the key to preparing the next-generation flexible battery.
